# Enhancement of Glycosaminoglycan-Rich Matrix Production in Human Marrow-Derived Mesenchymal Stem Cell Chondrogenic Culture by Lithium Chloride and SB216763 Treatment

**Published:** 2011-08-24

**Authors:** Mohamadreza Baghaban Eslaminejad, Negar Karimi, Maryam Shahhoseini

**Affiliations:** 1. Department of Stem Cell and Developmental Biology, Cell Science Research Center, Royan Institute for Stem Cell Biology and Technology, ACECR, Tehran, Iran; 2. Developmental Biology Department, University of Science and Culture, Tehran, Iran; 3. Department of Genetic, Reproductive Biomedicine Center, Royan Institute for Reproductive Biomedicine, ACECR, Tehran, Iran

**Keywords:** Human Mesenchymal Stem Cells, Lithium Chloride, Surface Marker, Chondrogenesis

## Abstract

**Objective:**

Cartilage mass produced from mesenchymal stem cell (MSC) differentiation would be a suitable candidate for use in regenerative medicine. Since the proper function of cartilage tissue is largely dependent on matrix glycosaminoglycan (GAG) contents, the objective of this study was to investigate the enhancing effect of two GSK3 inhibitors on the GAG content of cartilage produced by human marrow MSCs in vitro chondrogenesis.

**Materials and Methods:**

MSCs that were used in this experimental study were derived from human marrow aspirates and confirmed using standard assays. Optimal concentrations of Lithium chloride and SB216763 were determined based on the yield of viable cell numbers in MSC cultures treated with varying concentrations of either Lithium chloride or SB216763. Passaged-3 MSCs were then centrifuged into small aggregates and provided with a chondrogenic medium supplemented with either lithium or SB216763 reagent at the optimal concentration determined in the previous experiment. Three weeks after, GAG contents of the culture were quantified and compared to each other and the control.

**Results:**

According to our data, the cultures treated with 5 mM Lithium and 1 µM SB216763 tended to have comparatively more viable cells; therefore these concentrations were used in the differentiation experiments. The addition of either SB216763 or lithium to chondrogenic cultures appeared to significantly enhance cartilage matrix production. In SB216763 and Lithium-treated cultures average GAG concentrations were 6.17 ± 0.7 and 6.12 ± 1.1 µg/ml compared to 2.00 ± 0.3 µg/ml in the control (p<0.05).

**Conclusion:**

Using SB216763 and Lithium as supplements in human marrow MSC chondrogenic culture can lead to the production of cartilage mass high in GAG content.

## introduction

Mesenchymal stem cells (MSc) are classified as adult stem cells which reside in multiple tissues of the body. These cells were first isolated by Friedenstein et al from bone marrow tissues. These investigators described the cells as an adherent population with colonogenic ability capable of producing bone and cartilage-like deposits in cultures (1, 2). The capacity of self renewal over a relatively long period and the potential for multilineage differentiation, especially into bone cartilage and adipose cells, are the most important characteristics described for MSCs (3-5). Although no single specific surface antigen has been described to date for these cells, it has been shown that MSCs are characterized by not being positive to endothelial and hematopoietic markers but positive for certain markers including CD105, CD44, CD73 and CD90 (6).

MSCs are considered as appropriate candidates for implementing regeneration, especially in large articular cartilage defects, thanks to their multilineage differentiation potential and long term self renewal capacity (7-12). In such cell-based treatment of tissue defects one strategy would be the transplantation of fully differentiated MSCs into injured tissues. This requires designing appropriate culture conditions to differentiate the cells into the desired cell lineages.

There are two critical requirements to direct MSCs toward cartilage differentiation: the cells must be in close association with each other (condensation) and they should be provided with inducing molecules. The micro mass culture system has been designed to fulfill the first requirement (cell condensation). In this system, the cells are placed in a tube and centrifuged to a condensed aggregate. The resultant pellet is then added to a chondrogenic medium which provides appropriate inducers for cell differentiation. Transforming growth factor beta 3 (TGF-beta 3) is the most crucial inducer included in the chondrogenic medium. This growth factor initiates its own signaling pathway, resulting in the expression of cartilage specific genes and production of cartilage-specific matrix among the differentiated cells (13-17). Cartilage matrix is a flexible gel-like material which, in articular cartilage, acts as a biochemical spring in that it loses water when bearing weight and regains it upon weight removal. The interstitial fluid binding capacity of the cartilage matrix largely depends on its glycosaminoglycan (GAG) contents (18).

Some evidence has indicated the indirect effect of proteoglycan on cartilage development. In this regard Fisher et al have established a micro mass culture using limb mesenchymal cells treated with exogenous heparin sulfate proteoglycan. Their findings have indicated that exogenous heparin sulfate dramatically enhanced the ability of bone morphogenetic protein 2 (BMP2) to stimulate chondrogenesis and cartilage specific gene expression and reduce the concentration of BMP2 needed to stimulate chondrogenesis (19). In addition, the integrity as well as the proper function of the cartilage tissue is related to its GAG contents. Some investigations have emphasized the important role of cartilage GAG in developing osteoarthritis. According to this research, during the progression of osteoarthritis, it is the decreased proteoglycan content and altered proteoglycan structure of cartilage which finally lead to microscopic degenerative changes including cartilage cleft, chondrocyte cloning, loss of methachromasia and chondrocyte death (20-21).

The Wnt signaling pathway is a conserved molecular mechanism in multicellular animals regulating cell proliferation, differentiation, apoptosis, migration and cell fate (22). This pathway is initiated by Wnt molecules which, upon bonding on their membrane receptor, activate a mechanism resulting in expressions of target genes in the nucleus. It has been reported that the wnt signaling pathway may have an impact on cartilage differentiation. On the other hand, lithium Chloride and a reagent called SB216763 have been recognized as Glycogen synthase kinase 3 (GSK3) inhibitors of the Wnt signaling pathway (22-24). It is not clear whether or not Lithium and SB216763 are involved in MSC chondrogenesis in vitro. The present study deals with this issue.

The purpose of the present study is to investigate the chondrogenic effects of Lithium chloride and SB216763 on human marrow-derived MSCs in culture. To evaluate these effects, micromass chondrogenic cultures of human marrow-derived MSCs were established and treated with either Lithium or SB216763, followed by quantification of the GAG contents of either culture in comparison to that for conventional chondrogenic culture.

## Materials and Methods

### Isolation and culture of mesenchymal stem cells

This study was performed after being approved by the Ethics Committee of the Royan Institute. MSCs were derived from bone marrow aspirates collected from patients who volunteer for autologous MSC transplantation for articular cartilage lesions at the cell therapy centre of the Royan Institute. In brief, about 5 ml aliquots of bone marrow aspirate were diluted with 5 ml phosphate buffer solution (PBS), (Sigma, Germany), loaded over 20 ml of 1.077 g/ cm Lymphodex (Inno-Train, Sweden) and centrifuged at 400× g for 20 minutes. The layer containing mononuclear cells was then collected, washed two times with PBS and plated at 10^6^ cells/ml in dulbecco modified eagle medium (DMEM, Sigma, Germany) supplemented with 15% fetal bovine serum (FBS, Gibco, Germany) and 100 IU/ml penicillin and 100 µg/ml streptomycin (Gibco, Germany). The cultures were incubated at 37 ºC in an atmosphere of 5% CO_2_. After 3 days the culture medium was removed along with non adherent cells and fresh medium was added. The cultures were maintained till 70-80% confluency was achieved. At this time they were trypsinized using 0.05% Trypsin in 0.53 mM EDTA and replated at 1:3 ratios as passage 1. This procedure was repeated several times till sufficient cells were provided for the following experiments.

### Flowcytometry analysis

To determine the surface epitope profile of the isolated cells, flow cytometric analysis was performed. About 10^6^ cells from passaged-3 cultures placed in 5 ml tubes were provided with 5 µl of either propidium iodide (PI) or fluorescein isothiocyanate (FITC)-conjugated antibody and 5µl of blocking buffer and incubated at 4℃ for 20-25 minutes in a dark place. The cells were added with 1 ml washing buffer consisting of PBS supplemented with 1% FBS and centrifuged at 1200 rpm. The pellet was suspended in 300-500µl washing buffer and analyzed by flow cytometery equipment (FACScalibur cytometer equipped with 488 nm argon lasers). In this study IGG2 and IGG1 were used as isotope controls. WinMDI software was used to analyze the flow cytometric results. The following antibodies were used to stain the cells: FITC-conjugated CD31, CD33, CD90, CD105 and PE-conjugated CD11b, CD34, CD44, and CD73 (all purchased from Becton Dickenson, USA).

### Multilineage differentiation

#### Osteogenesis

To verify whether or not the isolated cells were populations of MSCs, differentiation cultures were established. The cells within passage 3 were plated in 6-well culture plates in a DMEM medium supplemented with 15% FBS and antibiotics at a density of 105cells/ml and allowed to become 70-80% confluent. For differentiation, the medium was then replaced by the induction medium; which was DMEM supplemented with 50 mg/ml ascorbic 2-phosphate (Sigma, USA), 10 nM dexamethasone (Sigma, USA) and 10 mM β glycerole phosphate (Sigma, USA), for about 3 weeks at the end of which the occurrence of differentiation was evaluated by Oil red staining as well as the RT-PCR method.

#### Alizarin red staining

The osteogenic cultures were first fixed with methanol for 10 minutes, followed by exposure to alizarin red solution for 2 minutes. Finally the cultures were washed with distilled water and observed using a light microscope.

#### Adipogenesis

For this purpose the medium of the partially confluent MSC cultures was replaced with adipogenic medium consisting of DMEM medium containing 100 nM dexamethazone (Sigma, USA) and 50 mg/ml indomethasine (Sigma, USA) for a 3-week culture period. Lipid droplets that developed in differentiating cells were then stained with Oil red staining.

#### Oil red staining

First the adipogenic cultures were fixed with 4% formalin at room temperature, followed by washing with 70% ethanol. Then the cultures were stained with oil red solution in 99% isopropanol for 15 minute. At the end, the stain solution was removed and the cultures were washed with 70% ethanol and observed by light microscope.

#### Chondrogenesis

For chondrogenic differentiation, a micromass culture system was used. About 2.5×10^5^ passaged-3 cells were aggregated into pellets by centrifuging at 1200 g for 5 minute then provided with a chondrogenic medium which consisted of DMEM supplemented with 10 ng/ml transforming growth factor-β3 (Sigma, USA), 10 ng/ml bone morphogenetic protein-6 (Sigma,USA), 50mg/ml insulin/ transferin/selenium+ premix (Sigma, USA) and 1.25 mg bovine serum albumin (Sigma, USA), and 1% FBS (Gibco, UK). Methachromatic matrices produced among the cartilage cells were visualized by toloidine blue staining of 5 µm sections prepared from the micromass cultures.

#### Toluidine blue staining

Chondrogenic pellets were fixed in 4% formalin; dehydrated in ascending ethanol; cleared in xylene; embedded in paraffin wax and sliced into 5µ sections by microtome. The sections were then stained with toluidine blue for 30 seconds at room temperature and viewed by light microscope.

#### RNA extraction and RT-PCR analysis of gene expression

Using RNX-Plus^TM^ solution (CinnaGen Inc., Tehran, Iran) total RNA was collected from the osteoblastic, chondrocytic and adipocytic cultures. The RNA samples were then digested with DNase I (Fermentas, Germany) to remove contaminating genomic DNA. Standard reverse-transcription reaction was performed with 5 µg total RNA using Oligo (dT) 18 as a primer and RevertAid ^TM^ H Minus First Strand cDNA Synthesis Kit (Fermentas, Germany) according to the manufacturer’s instructions. Subsequent PCR was done with 2.5 µl cDNA, 1X PCR buffer (AMS), 200 µM dNTPs, 0.5 µM of each primer pair (Table 1) and 1 unit/25 µl reaction Taq DNA polymerase (Fermentas, Germany). The products were analyzed on 2% agarose gel and visualized by ethidium bromide staining. For every reaction set, one RNA sample was prepared without RevertAidTMM-MuLV Reverse Transcriptase (RTreaction) in order to provide a negative control in the subsequent PCR. To minimize variation in the RT reaction, all RNA samples from a single experimental setup were reverse transcribed simultaneously.

### Lithium and SB216763 concentration selection

Varying concentrations of either Lithium Chloride (Sigma, USA) or SB216763 (Sigma, USA) were investigated in terms of their cytotoxic effects on human marrow-derived MSCs to determine the optimal concentration for use in chondrogenic cultures.

**Table 1:** Primers used in **RT-PCR**


Genes	Primer sequences (5'-3')	Annealing temperature (℃)	Length bp	Gene bank code
beta actin	F:5'tcc ctg gag aag agc tac g3'	60	131	NM-00110103
	R:5'gta gtt tcg tgg atg cca ca 3'			
Osteocalcin	F:5'ggcagcgaggtagtgaagag3'	56	196	NM-199173.3
	R:5'cagcagagccacaccctagac3'			
COL IA	F:5'gtg gtg aca agg gtg aga cac3'	62	225	NM-000088
	R:5'caa cag gac cag cat cac cag3'			
PPARα	F:5'tgc tat cat ttg ctg tgg ag3'	60	175	NC_000022.10
	R:5'act ccg tct tct tga tga t3'			
PPARॕ	F:5'cta aag agc ctg cga aag 3'	60	186	NC_000003.11
	R:5'tgt ctg tct ccg tct tct tg3'			
COL2A	F:5'tct acc cca atc cag caa ac 3'	60	170	NC_000012.11
	R:5'gcg tag gaaggt cat ctg ga3'			
Aggrecan	F:5'ctg gac aag tgc tat gcc g 3'	58	191	NC_000015.9
	R:5'gaa gga acc gct gaa atg c3'			


For this purpose 2×10^4^ passaged-3 cells were plated in a 24-well culture plate in a DMEM medium containing 15% FBS and antibiotics. After 24 hours the medium was supplemented with either varying concentrations of Lithium, including 3, 5 and 7 mM, or different concentrations of SB216763, including 0.2, 0.1, 1, 2 and 3 µM. All cultures were maintained for an additional 24 hours at the end of which the cultures were evaluated for the number of viable cells.

### MTT assay

To determine the viable cell number in each culture, an MTT [3-(A, 5-dimethylthiazol-2-yl)-1, 5-diphenyl tetrazulium bromide] (MTT, Sigma, USA) assay was performed. The cells were washed with PBS, provided with MTT (5 mg/mL in PBS) solution diluted in DMEM at a ratio of 1:5 and incubated for 2 hours at 37ºC. The MTT was then replaced by 0.5 ml of extraction solution (dimethylsulphoxide: DMSO). The absorbance of the supernatant was then recorded using a microplate reader (BioTek EL ×800, USA) at 540 nm. The cell number was determined through a standard curve that was established by using a known cell number.

### Chondrogenic culture with Lithium chloride treatment

Multiple pellets of MSCs were produced by centrifuging about 2×10^5^ passaged-3 cells at 1200 rpm. Some pellets were then cultivated in chondrogenic medium (DMEM contained 10 ng/ml transforming growth factor-β3, 10 ng/ml bone morphogenetic protein-6, 50mg/ml insulin/ transferin/selenium+ premix and 1.25 mg bovine serum albumin, and 1% fetal bovine serum) supplemented with 5 mM Lithium Chloride and maintained at 37 ºC in an atmosphere of 5% CO_2_ and 95% humidity for a period of about 21 days, during which the medium was refreshed twice weekly. The pellets cultivated in chondrogenic medium without any treatment were taken as the controls. At the end of cultivation period some pellets were sectioned for toluidine blue staining and the others were utilized for the quantification of their GAG contents.

### Chondrogenic culture with SB216763 treatment

To establish this culture, similar MSCs pellets were provided with chondrogenic medium supplemented with 1 µm SB216763 and incubated at conditions similar to that for the Lithium-treated cultures.

### Quantification of GAG

Three weeks after initiation of the chondrogenic culture, the GAG deposited among the differentiating cells was quantified using an acidic mucopolysaccarid kit. The procedure was performed according to the manufacturer’s instructions. Briefly, enzyme solution provided with the kit was added to the cells, followed by heating at 60ºC for an hour. After cooling, the digested tissue was pipetted into a 5 ml tube together with about 2 ml of staining solution. The solution was mixed thoroughly for 20min and the absorption value obtained at 650nm using an Elisa plate reader was recorded. To calculate the amount of GAG in each culture the optical density (OD) values recorded for each group were compared with the calibration curve that was plotted by graphing absorbance as a function of known concentrations of chondroitin sulfate that were provided with the kit.

### Statistical Analysis

Each experiment described in this study was replicated for cells from 5 human beings. All values are stated as means ± standard deviations. The results of the SB216763 and Lithium concentration selection as well as the data from the GAG quantification were analyzed with ANOVA. A p<0.05 was considered to be statistically significant.

## Results

### Cell culture

**Fig 1:**
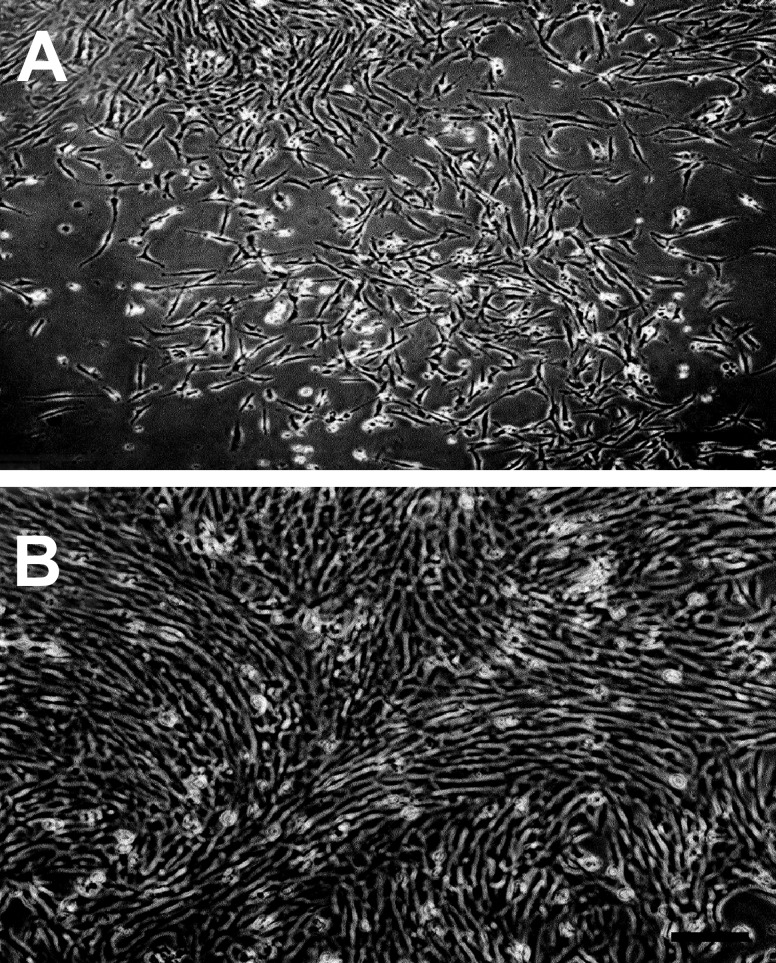
Human marrow mononuclear cell culture. A. five days after culture initiation: several colonies consisting of elongated fibroblastic cells can be seen. B. The colonies grew larger and eventually became confluent. The cells still maintained their fibroblastic morphology (Bar= 100 µm).

At primary culture, some marrow cells adhered and established the culture while others failed to adhere, remained floating and were later discarded when the medium was replaced. Adhered cells formed several small colonies. These colonies grew larger and finally became confluent within 2 weeks (Fig 1A, B). The cells were observed to be fibroblastic in morphology at primary culture. This fibroblastic morphology was maintained at subsequent subcultures.

### Flow cytometry

**Fig 2:**
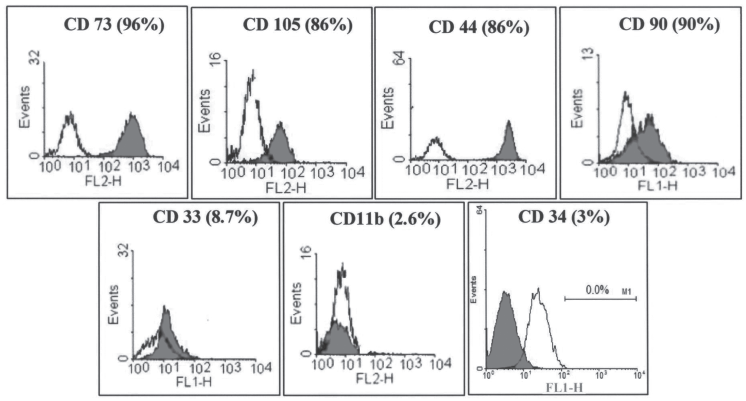
Flow cytometric analysis for some surface markers on human marrow mesenchymal stem cells. A majority of the cells were positive to **CD73, CD105, CD44 and CD90. CD33, CD11b** and **CD34** were only expressed on a minority of the cells.

According to the flow cytometry results, about 90% of the passaged-3 human MSCs expressed CD73, CD44, CD90 and CD105 on their surfaces. The surface markers CD33, CD34 and CD11b tended to be expressed on a very small percentage of the cells examined (Fig 2).

**Fig 3:**
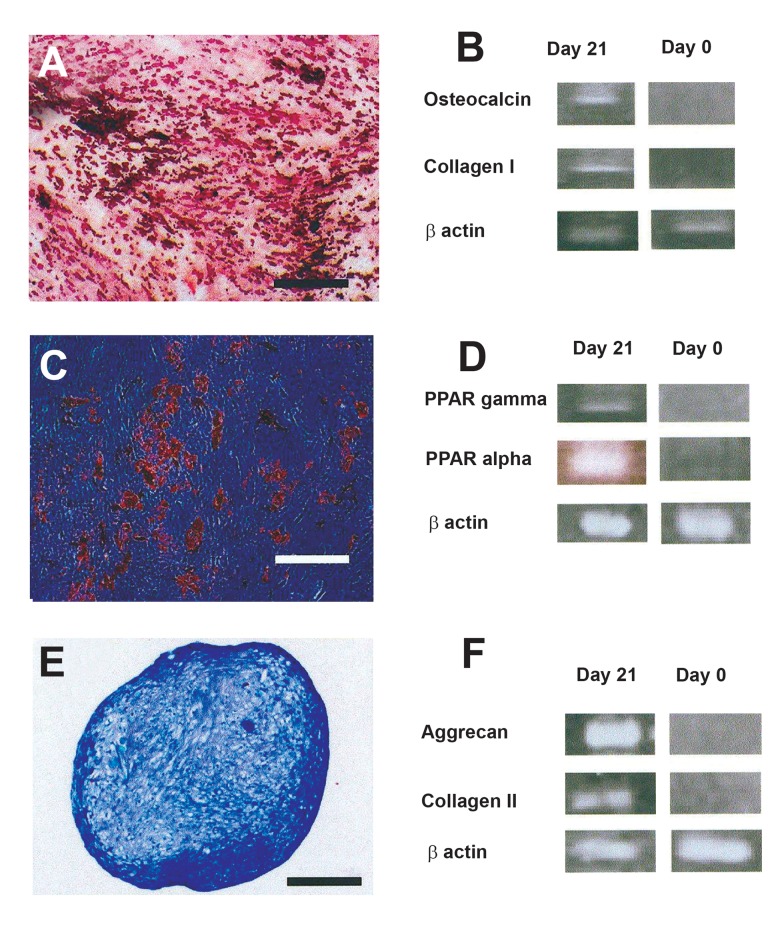
Multilineage differentiation of the passaged-3 human mesenchymal stem cells. **A-B**) osteogenic differentiation culture was positively stained red with alizarin red. In this culture bone specific genes including osteocalcin and collagen **I** were expressed. **C-D**) Lipid droplets in adipogenic culture appeared red upon oil red staining. **PPAR** gamma and **PPAR** alpha were expressed in adipocytic cells. **E-F**) the sections from micro mass culture for cartilage differentiation were metachromatically stained purple with toloidine blue. Cartilage related genes including aggrecan and collagen **II** were expressed at chondrogenic culture (Bar= 500 µm)

### Multilineage differentiation

Osteogenic culture: Small nodule-like aggregations developed in the osteogenic cultures and stained red upon alizarin red staining (Fig 3A). RT-PCR analysis indicated that the mRNA of bone specific proteins including osteocalcin, and collagen I was produced in the culture (Fig 3B).

Adipogenic culture: Lipid droplets developed in the differentiating cells in the adipogenic cultures and were positively stained red with oil red staining used for adipocyte detection (Fig 3C). RT-PCR analysis further confirmed adipogenesis by revealing the expression of adipocyte marker genes, including PPAR-alpha and PPAR-gamma in the cultures (Fig 3D).

Chondrogenic culture: Toluidine blue staining of the sections from micro mass cultures indicated cartilage matrix production in the chondrogenic cultures (Fig 3E). RT-PCR analysis revealed the production of collagen II and aggrecan mRNA in the differentiated cells (Fig 3F).

### Lithium and SB216763 concentration selection

**Fig 4:**
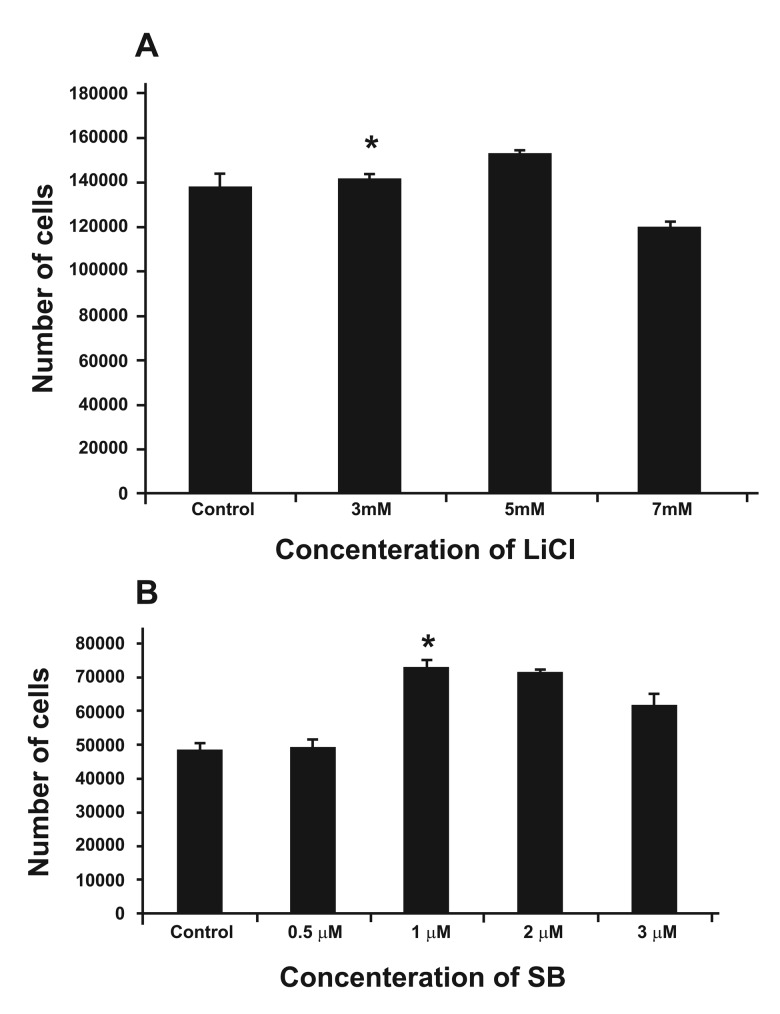
Treatment of the human marrow mesenchymal stem cell culture with varying concentrations of Lithium and SB216763. A. Graph indicating that 5 mM Lithium is associated with a significantly higher density of viable cells. * indicates a significant difference, p<0.05. B. Graph indicating that treatment of the culture with 1 µM **SB216763** resulted in a relatively higher density of viable cells. * indicates a significant difference, p<0.05.

According to the MTT results, 5 mM lithium chloride appeared to be the more optimal concentration in that significantly more viable cells (1.52×10^5^ ± 0.021×10^5^) were found (p<0.05). The mean number of viable cells for 3 and 7 mM Lithium Chloride and for the control were 1.41×10^5^ ± 0.026×10^5^, 1.18×10^5^ ± 0.027×10^5^ and 1.37×10^5^ ± 0.062×10^5^ respectively (Fig 4A). Regarding the SB216763 MTT results, 1 µm concentration appeared to be the best, since this concentration resulted in the production of significantly more viable cells (7.29×10^4^ ± 0.006×10^4^), p<0.05. The mean number of viable cells for other concentrations, including 0.2, 0.5, 2, 3 and the control were 4.90×10^4^ ± 0.023×10^4^, 4.90×10^4^ ± 0.023×10^4^, 7.13×10^4^ ± 0.034×10^4^, 6.14×10^4^ ± 0.018×10^4^, 4.87×10^4^ ± 0.019×10^4^ respectively (Fig 4B).

### Chondrogenic cultures treated with Lithium chloride

**Fig 5:**
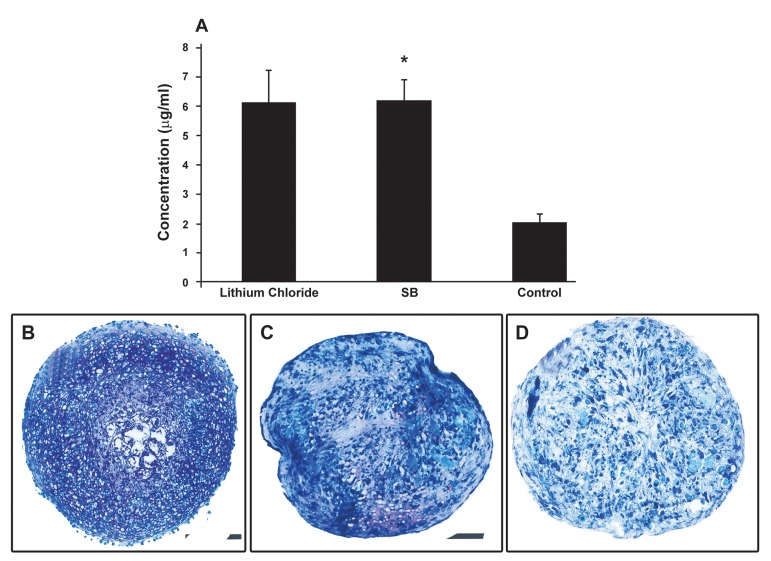
Human marrow-derived **MSC** chondrogenic cultures treated with either **SB216763** or Lithium chloride. **A**. The graph demonstrating that in a chondrogenic culture treated with **SB216763** (**SB**) and Lithium there was significantly more **GAG** in the **ECM**. * Indicated a significant difference, p<0.05. Representative sections from the cultures treated with Lithium chloride (**B**) and **SB216763** (**C**). Apparently, abundant methachromatic matrix was produced in these cultures compared to that in the control untreated culture (**D**). All sections were stained with toluidine blue (Bar = 500 µm)

According to observations made on the sections stained with toluidine blue, human MSC chondrogenic cultures treated with lithium chloride appeared to contain metachromatic matrix, indicating that the cells had been converted into chondrocytic cell lineages and deposited cartilage-specific matrix rich in GAG (Fig 5A). In this regard the cultures appeared to have more methachromatic matrix than the control (untreated) culture (Fig 5C).

### Chondrogenic cultures treated with SB216763

Similarly, sections prepared from the cultures treated with SB216763 contained methachromatic matrix which stained purple with toluidine blue (Fig 5B). Apparently, in this regard, there was no significant difference between the SB216763 and Lithium treated cultures but the difference between SB216763 treated cultures and the control, untreated culture was obvious.

### GAG quantification

The quantification of GAG in the cultures confirmed observations made on the stained sections prepared from different culture groups. According to this assay the GAG concentration for the culture with SB216763 supplementation was about 6.17 ± 0.7 µg/ ml (Fig 5A). In this regard there was no significant difference between SB216763 and Lithium-treated cultures (6.12 ± 1.1µg/ml). The GAG concentration of both cultures were significantly higher than that of the controls (2.00 ± 0.3µg/ml; p<0.05).

## Discussion

The present investigation was an attempt to enhance in vitro chondrogenic differentiation of human marrow-derived MSCs. Cartilage tissue produced by MSC differentiation would be an appropriate candidate with which to implement regeneration of articular cartilage, which proves hard to repair. The alternative option, i.e. chondrocyte transplantation, is associated with problems such as difficult *ex vivo* propagation of the cells and their dedifferentiation during proliferation. One important strategy in cell-based treatment of tissue defects is transplantation of fully-differentiated, rather than undifferentiated cells. This requires an appropriate design for in vitro cartilage differentiation of the MSCs, with suitable medium supplemented by a potent inducer. In the present study, to enhance chondrogenic effects, two agents that inhibit GSK3 were added to conventional chondrogenic medium. Our results indicated that both agents were able to enhance cartilage differentiation in human MSCs, reflected in a significantly higher production of GAG among the differentiated cells. These data will be of great importance to professionals involved in a cell therapy approach to regenerating cartilage defects and enable them to generate a cartilage mass with large GAG contents.

In a former study, Lithium Chloride was used in chondrogenic cultures of MSCs, which had also been treated with TGF beta3 growth factor. The objective of the investigation was to stimulate the Wnt signaling pathway and to examine the collaboration of this pathway with the TGF beta pathway. Therefore the authors investigated the expression of certain molecules, including beta catenin which is involved in the signaling pathway (25). The present study was designed to explore the Lithium chloride effects on matrix production in human marrow MSC chondrogeenic culture. To achieve this goal, the culture was treated with Lithium chloride, followed by quantification of GAG production among the differentiating cells. Regarding SB216763, no data were available on the chondrogenic effect of this reagent on MSC cartilage differentiation culture. Previous research work has indicated the role of SB216763 as a GSK3 inhibitor in chromaffin cells found in the adrenal gland (26).

A limited number of studies have also addressed the effects of Lithium chloride and SB216763 reagents on chondrocyte differentiation in some non MSC cell cultures. For example Ravi et al have conducted research to investigate the role of GSK3 inhibition on endochondral bone development. They have established an explant culture of murine metatarsal bone and treated the cultures with either lithium chloride or SB216763, two reagents with potent GSK3 inhibitory activity (27). The following evaluations of the culture demonstrated that chondrocyte differentiation was repressed and the cell proliferation was inhibited in explants. In the present study, in which human MSCs were cultivated in a micro mass system in the presence of lithium chloride or SB216763, the results tended to be opposite, in that we observed the enhancement of cartilage differentiation (manifested as increased GAG production). The causes of such discrepancies are probably the differences in cell kind, as well as the culture conditions utilized by each study. More notably, in contrast to the medium used by Ravi et al, our culture contained TGF beta3 in addition to either the lithium or SB216763 reagents. It would be the interaction or cross-talk between the pathways initiated by TGF beta3 and either Lithium chloride or SB216763 that finally resulted in the enhanced chondrogenesis observed in the present study. This point has already also been suggested by Nemoto et al. (26). In another study, conducted by Kitton et al, similar results have been obtained using bovine retinal pericyte micro mass cultures treated with a chondrogenic medium supplemented by both TGF beta3 and Lithium chloride (28).

In this study one major concern was the selection of appropriate concentrations of Lithium and SB216763 for use in chondrogenic culture. There were two potential options: one was to examine several concentrations of each reagent directly in chondrogenic cultures and the alternative was to first examine a range of concentrations of both reagents in proliferation cultures in order to determine concentrations with minimal cytotoxic effects and then to treat the differentiation culture with those concentrations. Of these potential options we used the second, since examining the varying concentrations of the reagents directly in chondrogenic medium requires many more cells, expensive chondrogenic media and expensive GAG quantification kit. Given the human source of the cells, obtaining sufficient cell numbers requires more initiating material (bone marrow). On the other hand it is more likely that the concentration of reagents apparently associated with a relatively less viable cell number in proliferation culture may have a more potent chondrogenic effect. This point, however, needs further investigation.

The other point that needs to be explained is the mechanism by which these two reagents promote their effects in MSC chondrogenic culture. Bearing in mind the role of both reagents as GSK3 inhibitors helps shed light on the possible course of events occurring inside differentiating cells. GSK3 inhibition can block destruction by Axin/ APC/GSK3, hence beta catenin is protected from destruction and finally enters the cell nucleus to exert its effects on target transcription factors. The end result of such events is the production of cartilage mass high in GAG content, which accumulates among the differentiating cells as ECM. The regulation of the molecules involved in the Wnt pathway was not examined in this study and therefore needs to be confirmed by further investigation.

## Conclusion

Taken together, both lithium chloride and SB216763 treatment of chondrogenic culture prepared from human marrow-derived MSCs produced a statistically significant greater yield of GAG-rich extracellular matrix (ECM). Using these reagents in chondrogenic cultures can lead to the production of cartilage mass high in GAG content. Since GAG imparts the specific property of cartilage tissue i.e. load bearing without tissue disassociation, such cartilage would be a suitable construct to implement regeneration of articular cartilage defects.

## References

[1] Friedenstein AJ, Piatetzky-Shapiro II, Petrakova KV (1966). Osteogenesis in transplants of bone marrow cells. J embryol Exp Morphol.

[2] Friedenstein AJ, Chailakhjan RK, Lalykina KS (1970). The development
of fibroblast colonies in monolayer cultures
of guinea-pig bone marrow and spleen cells. Cell Tissue Kinet.

[3] Eslaminejad MB, Eftekhari-Yazdi P Mesenchymal
stem cells: In vitro differentiation among bone and
cartilage cell lineages. Yakhteh.

[4] Eslaminejad MB, Talkhabi M, Zainali B (2008). Effect of Lithium
chloride on proliferation and bone differentiation
of rat marrow-derived mesenchymal stem cells in culture. Iranian J Basic Med Sci.

[5] Prockop DJ (1997). Marrow stromal cells as stem cells
for nonhematopoietic tissues. Science.

[6] Dominici M, Le Blanc K, Mueller I, Slaper-Cortenbach I, Marini F, Krause D (2006). Minimal criteria for defining
multipotent mesenchymal stromal cells. The international societry for cellular therapy position statement. Cytotherapy.

[7] Barry FP (2003). Mesenchymal stem cell therapy in joint disease. Novartis Found Symp.

[8] Horwitz EM, Gordon PL, Koo WK, Marx JC, Neel MD, McNall RY (2002). Isolated allogenic bone marrow-
derived mesenchymal cells engraft and stimulate
growth in children with osteogenesis imperfecta:
Implications for cell therapy of bone. Proc Natl Acad Sci USA.

[9] KoÇ ON, Gerson SL, Cooper BW, Dyhouse SM, Haynesworth SE, Caplan AI (2000). Rapid hematopoietic
recovery after confusion of autologous-blood stem
cells and culture-expanded marrow mesenchymal
stem cells in advanced breast cancer patients receiving
high dose chemotherapy. J Clin Oncol.

[10] Petite H, Viateau V, BensaÏd W, Meunier A, de Pollak C, Bourguignon M (2000). Tissue engineered bone regeneration. Nat Biotechnol.

[11] Quarto R, Mastrogiacomo M, Cancedda R, Kutepov SM, Mukhachev V, Lavroukov A (2001). Repair of large
bone defect with the use of autogenic bone marrow
stromal cell. N Engl J Med.

[12] Grinnemo KH, Månsson A, Dellgren G, Klingberg D, Wardell E, Drvota V (2004). Xenoreactivity and engraftment
of human mesenchymal stem cells transplantated
into infarcted rat myocardium. J Thorac Cardiovasc Surg.

[13] Johnstone B (2002). Mesenchymal stem cells and chondrogenesis. Eur Cell Mater.

[14] Bosnakovski D, Mizuno M, Kim G, Ishiguro T, Okumura M, Iwanaga T (2004). Chondrogenic differentiation of
bovine bone marrow mesenchymal stem cells in pellet
culture system. Exp Hematol.

[15] Indrawattana N, Chen G, Tadokoro M, Shann LH, Ohgushi H, Tateishi T (2004). Growth factor combination
for chondrogenic induction from human mesenchymal
stem cell. Biochem Biophys Res Commun.

[16] Eslaminejad MB, Nikmahzar A, Thagiyar L, Nadri S, Massumi M (2006). Murine mesenchymal stem cells isolated
by low density primary culture system. Develop Growth Differ.

[17] Eslaminejad MR, Nikmahzar A, Piriea A, Eftekhari yazdi P (2006). The structure of Human Mesenchymal Stem
Cells differentiated into cartilage in micro mass culture
system. Yakhteh.

[18] Junqueira LC, Carneiro J, Kelley RO (1992). Basic histology.

[19] Fisher MC, Li Y, Seghatoleslami MR, Dealy CN, Kosher RA (2006). Heparan sulfate proteoglycans including
syndecan-3 modulate BMP activity during limb cartilage
differentiation. Matrix Biol.

[20] Horton WE Jr, Yagi R, Laverty D, Weiner S (2005). Overview
of studies comparing human normal cartilage with
minimal and advanced osteoarthritic cartilage. Clin Exp Rheumatol.

[21] Mandelbaum B, Waddell D (2005). Etiology and pathophysiology
of osteoarthritis. Orthopedics.

[22] Hoppler S, Kavanagh CL (2007). Wnt signalling: variety at
the core. J cell sci.

[23] Stambolic V, Ruel L, Woodgett JR (1996). Lithium inhibits
glycogen synthase kinase-3 activity and mimics
wingless signalling in intact cells. Curr Biol.

[15] Coghlan MP, Culbert AA, Cross DA, Corcoran SL, Yates JW, Pearce NJ (2000). Selective small molecule
inhibitors of glycogen synthase kinase-3 modulates
glycogen metabolism and gene transcription. Chem Biol.

[25] Zhou S, Eid K, Glowacki J (2004). Cooperation between
TGF-beta and Wnt pathways during chondrocyte and
adipocyte differentiation of human marrow stromal
cells. J Bone Miner Res.

[26] Nemoto T, Kanai T, Yanagita T, Satoh S, Maruta T, Yoshikawa N (2008). Regulation of Akt mRNA and protein
levels by glycogen synthase kinase-3beta in adrenal
chromaffin cells: effects of LiCl and SB216763. Eur J Pharmacol.

[27] Kapadia RM, Guntur AR, Reinhold MI, Naski MC (2005). Glycogen synthase kinase 3 controls endochondral
bone development:Contribution of fibroblast growth
factor 18. Dev Biol.

[28] Kirton JP, Crofts NJ, George SJ, Brennan K, Canfield AE (2007). Wnt/ β-Catenin Signaling Stimulates Chondrogenic
and Inhibits Adipogenic Differentiation of Pericytes:
Potential Relevance to Vascular Disease?. Circ Res.

